# Fusion gene map of acute leukemia revealed by transcriptome sequencing of a consecutive cohort of 1000 cases in a single center

**DOI:** 10.1038/s41408-021-00504-5

**Published:** 2021-06-16

**Authors:** Xue Chen, Fang Wang, Yang Zhang, Xiaoli Ma, Panxiang Cao, Lili Yuan, Lan Wang, Jiaqi Chen, Xiaosu Zhou, Qisheng Wu, Ming Liu, David Jin, Hongxing Liu

**Affiliations:** 1 Division of Pathology & Laboratory Medicine, Hebei Yanda Lu Daopei Hospital, 065201 Langfang, China; 2 Beijing Lu Daopei Institute of Hematology, 100176 Beijing, China; 3 Division of Pathology & Laboratory Medicine, Beijing Lu Daopei Hospital, 100176 Beijing, China

**Keywords:** Acute myeloid leukaemia, Acute lymphocytic leukaemia

## Abstract

Fusion genes (FGs) are important genetic abnormalities in acute leukemias, but their variety and occurrence in acute leukemias remain to be systematically described. Whole transcriptome sequencing (WTS) provides a powerful tool for analyzing FGs. Here we report the FG map revealed by WTS in a consecutive cohort of 1000 acute leukemia cases in a single center, including 539 acute myeloid leukemia (AML), 437 acute lymphoblastic leukemia (ALL), and 24 mixed-phenotype acute leukemia (MPAL) patients. Bioinformatic analysis identified 792 high-confidence in-frame fusion events (296 distinct fusions) which were classified into four tiers. Tier A (pathogenic), B (likely pathogenic), and C (uncertain significance) FGs were identified in 61.8% cases of the total cohort (59.7% in AML, 64.5% in ALL, and 63.6% in MPAL). FGs involving protein kinase, transcription factor, and epigenetic genes were detected in 10.7%, 48.5%, and 15.1% cases, respectively. A considerable amount of novel FGs (82 in AML, 88 in B-ALL, 13 in T-ALL, and 9 in MPAL) was identified. This comprehensively described real map of FGs in acute leukemia revealed multiple FGs with clinical relevance that have not been previously recognized. WTS is a valuable tool and should be widely used in the routine diagnostic workup of acute leukemia.

## Introduction

Fusion genes (FGs) are major molecular biological abnormalities in acute leukemia, and all well-known FGs in leukemias are founder variations and play as crucial tumorigenesis factors. They exist stably with tumor cells and have been used as molecular markers for the diagnosis, classification, risk stratification, and targeted therapy of leukemia. They can also be used as molecular markers for monitoring minimal residual disease (MRD) with high sensitivity. Based on their essential role in leukemogenesis, the WHO classification of neoplastic diseases of the hematopoietic and lymphoid tissues has incorporated dozens of FGs as essential molecular markers since 2001 [1]. Ever since then, screening multiple common FGs simultaneously and then quantitatively monitoring the positive ones have been introduced into the routine clinical diagnostic workup of acute leukemia.

We have previously reported common FGs were presented in ~41% of acute myeloid leukemia (AML) and 29% of acute lymphoblastic leukemia (ALL) cases, respectively [2, 3]. The distribution of FGs in acute leukemia presented a typical long-tail phenomenon, which meant that several FGs with high-frequencies were followed by a large number of FGs with low-frequencies which gradually “tails off” asymptotically. The fusion events at the far end of the tail had a very low probability of occurrence. In AML, 23 kinds of distinct FGs were detected in 1292 of 3135 patients. Eight FGs with frequencies of more than 1% accounted for 94% of all positive FGs. The other 15 FGs with frequencies below 1% constituted the long tail of the distribution. Similarly, in ALL, only 5 FGs had positive rates of more than 1% and accounted for 89% of all positive FGs. The individual positive rates of dozens of FGs were all below 1%, even if they have been frequently reported in the literature [2, 3].

The rapid development of sequencing technology and the decline of sequencing costs in recent years have made whole transcriptome sequencing (WTS) more accessible, which can analyze known FGs and has unique advantages in identifying unknown rare and variant FGs. Several groups have discovered numerous novel FGs, such as those involving *ZNF384*, *MEF2D*, *PAX5*, and *DUX4* rearrangements, among cases that were once regarded as B-other-ALL with no defining cytogenetic abnormalities [4–9]. To better understand the incidences of FGs and their pathological characteristics, we proposed the conception of the “fusion gene family, FG-FM” to classify fusions that involve one protagonist gene and various fusion partners [10]. FGs in the same family often share commonalities in pathogenicity, clinical features, and treatment outcomes. Although most newly identified FGs are individually rare, the overall incidence is significant due to the wide variety. To date, the exact population of pathogenetically driver FGs undiscovered in acute leukemias and the total positive rate of them remains unknown. FGs with pathological significance, even the individually rare FGs, still have definite significance in clinical diagnosis, treatment guidance, and MRD monitoring. Thus, it is essential to decipher the distribution feature of FGs in acute leukemias and investigate effective detection methods.

Considering the versatility provided by WTS would uncover otherwise undetected FGs, we have started to use WTS to analyze FGs for accompanying diagnosis in our hospitalized acute leukemia patients since September 2018. Here, we provide the retrospective overview of the FG map in our patients.

## Subjects and methods

### Patients

From September 2018 to September 2020, a consecutive cohort of 1000 cases with confirmed diagnoses of acute leukemia in Hebei Yanda Lu Daopei hospital was enrolled in this study, including 405 children (≤18 years, median age eight years, range 8 months–18 years; 237 males, 168 females) and 595 adults (>18 years, median age 43 years, range 19–89 years; 320 males, 275 females). Among them, 539 were AML (137 children and 402 adults); 437 were ALL (257 children and 180 adults), including 365 B-ALL and 72 T-ALL; and 24 were mixed-phenotype acute leukemia (MPAL) (11 children and 13 adults). The diagnosis was made according to the 2016 revision to the WHO classification of tumors of hematopoietic and lymphoid tissues [11, 12]. Fifty healthy donors in Hebei Yanda Lu Daopei Hospital were included as controls. The study was approved by the medical ethics committees at Hebei Yanda Lu Daopei Hospital. Written informed consent for medical record review was obtained from all patients and healthy controls or their guardians following the Declaration of Helsinki.

### Sample preparation

Bone marrow samples were collected. Nucleated cells (1.0 × 10^7^) were used for genomic DNA extraction. Nucleated cells (5.0 × 10^6^) were used for total RNA extraction by the guanidinium thiocyanate-phenol-chloroform method using a TRIZOL reagent according to the manufacturer’s recommendations (Invitrogen Corporation, Carlsbad, CA, USA). Complementary DNA (cDNA) was synthesized using M-MLV Reverse Transcriptase (Promega Corporation, Madison, WI, USA) or a Maxima First Strand cDNA Synthesis Kit (Thermo Fisher Scientific, Inc., Waltham, MA, USA) according to the manufacturer’s protocol.

### FGs screening

All enrolled cases underwent common FGs screening parallelly. A total of 131 mRNA isotypes of 41 FGs (Table S1), which have been reported as recidivists in leukemia patients, were screened by multiplex-nested reverse-transcription PCR (RT-PCR) according to the protocols we previously reported [2, 3].

### Library preparation, WTS

Total RNA quality was assessed using NanoPhotometer spectrophotometer, Qubit 2.0 Fluorometer (Life Technologies), and Agilent 2100 Bioanalyzer. High quality RNA was then subjected to library preparation using a NEBNext^®^ Ultra^TM^ Directional RNA Library Prep Kit for Illumina^®^ (New England BioLabs) according to the manufacturer’s instructions with input ≥1 μg of total RNA. Paired-end sequencing with a read length of 150 bp was performed on Illumina HiSeq 2500 platform, yielding at least 50 (71.35 ± 11.10, mean ± s.d.) million sequence reads mapped to 16,052 ± 996 RefSeq entries per sample. Image analysis, base calling, and quality check were performed with Illumina data analysis pipeline RTA v1.18.64 and Bcl2fastq v1.8.4. The sequence reads were provided in compressed Sanger FASTQ format.

### FG detection in WTS data

We utilized the Arriba (v1.0.1) [13] algorithm for the detection of fusion transcripts in WTS data. To each prediction Arriba assigns a confidence of low, medium, or high. The confidence reflects three aspects, namely the likelihood that the transcript is aberrant (not seen in healthy tissue); it can be explained by an underlying genomic rearrangement; it is not an artifact. The number of supporting reads is one of the most helpful attributes to distinguish artifacts from true events. Arriba assumes a polynomial relationship between the number of supporting reads and the level of background noise. Only candidates with more supporting reads than the estimated level of background noise are reported. In addition, the statistical model of Arriba incorporates several covariates that correlate with the level of background noise, including the sequencing depth, the breakpoint distance, the library preparation protocol, and the location of the breakpoints.

In our analysis, we defined “positive fusion events” as in-frame FGs which were selected from the high-confidence predictions, unless Arriba annotated the events as ‘read-through’. Reciprocal fusion transcripts were counted as one fusion event. FGs were defined as “novel” if they were not reported in previous literature and not found by performing queries against the Atlas of Genetics and Cytogenetics in Oncology and Haematology (http://atlasgeneticsoncology.org/index.html), the Tumor Fusion Gene Data Portal (https://www.tumorfusions.org), and ChimerDB [14]. For the validation of the FGs not detected in FGs screening, RT-PCR was performed, followed by Sanger sequencing.

### Pathogenicity evaluation of FGs

We classified the final FGs list into four tiers based on our current understanding of their pathogenic impact: (A) pathogenic: well-known FGs or new members of common FG-FMs with definite pathogenicity in hematological malignancies or other tumors, e.g., *BCR-ABL1* or new members of *ABL1*-FM; (B) likely pathogenic: rarely reported FGs or new members of rare FG-FMs in hematological malignancies or other tumors without functional verification, e.g., *TBC1D15-RAB21*, which was reported in acute promyelocytic leukemia, but no functional verification was reported [15]; or one of the partner genes was reported in hematological malignancies in other forms of abnormalities, such as mutation, e.g., *ASXL2-ITSN2* (*ASXL2* is frequently mutated in AML patients [16]; (C) uncertain significance: both fusion partners not reported before in hematological malignancies in any form of genomic alterations, e.g., *ANAPC7 -GPN3*; and (D) non-pathogenic: FGs detected in normal samples.

### Statistical analysis

We used *χ*2 and Fisher’s exact tests to compare differences in frequencies of FGs between different age groups and leukemia subtypes. We performed all of the analyses with SPSS Statistics software, version 20 (IBM Corp., Armonk, New York, US). A two-sided *P*-value of <0.05 was considered to be statistically significant.

## Results

### Spectrum and incidence of FGs in all patients

We identified 792 high-confidence in-frame fusion events of 296 distinct FGs in 1000 acute leukemia cases. We further classified these FGs into four tiers based on pathogenicity investigation, and the number of tier A, B, C, and D FGs was 116 (39.2%), 114 (38.5%), 58 (19.6%), and 8 (2.7%), respectively. The 8 tier D FGs (39 total fusion events), which were unlikely to be pathogenic, were not analyzed further.

**Fig. 1:**
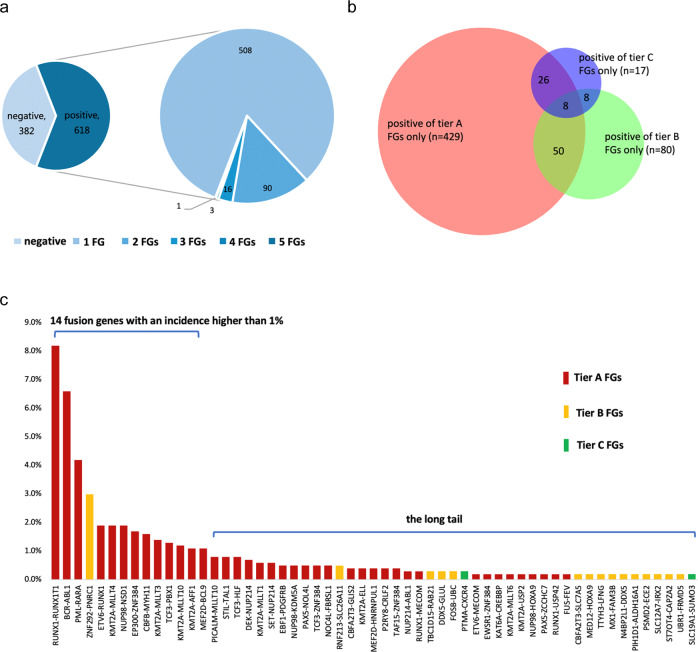
Tier A, B, and C fusion genes (FGs) detected in the 1000 acute leukemia cases. **a** Tier A, B, and C FGs were identified in 618 samples. Most of them showed 1 fusion event (n = 508), 90 patients harbored 2 fusions, and 16 patients had 3 fusions. Only 3 patients had 4 fusions and 1 patient had 5 fusions. **b** Venn diagram represented the pathogenicity evaluation of the FGs found in the 618 cases. **c** A total of 57 distinct recurrent FGs were identified, including 39 tier A, 16 tier B, and 2 tier C FGs, respectively. Fourteen FGs had incidences higher than 1%.

753 fusion events (525 tier A, 167 tier B, and 61 tier C, respectively) were identified in 618 (61.8%) samples. The majority of cases showed 1 fusion event (*n* = 508; 82.2%), 90 patients harbored 2 fusions (14.6%), and 16 patients had 3 fusions (2.6%). Only 3 patients had 4 fusions (0.5%), and 1 patient had 5 fusions (0.2%) (Fig. 1a).

Tier A, tier B, and tier C FGs were detected in 513 (51.3%), 146 (14.6%), and 59 (5.9%) cases, respectively. Concurrence of tier A and tier B fusions was detected in 50 (5.0%) cases; coexistence of tier A and tier C fusions was identified in 26 (2.6%) cases; co-occurrence of tier B and tier C fusions was found in 8 (0.8%) cases; and the remaining 8 (0.8%) cases had tier A and tier B and tier C fusions simultaneously (Fig. 1b).

We found 57 kinds of recurrent FGs that occurred at least twice, including 39 tier A, 16 tier B, and 2 tier C FGs, respectively. Fourteen FGs with relative high incidences were: *RUNX1-RUNX1T1* (8.2%), *BCR-ABL1* (6.6%), *PML-RARA* (4.2%), *ZNF292-PNRC1* (3.0%), *KMT2A-MLLT4* (1.9%), *NUP98-NSD1* (1.9%), *ETV6-RUNX1* (1.9%), *EP300-ZNF384* (1.7%), *CBFB-MYH11* (1.6%), *KMT2A-MLLT3* (1.4%), *TCF3-PBX1* (1.3%), *KMT2A-MLLT10* (1.2%), *KMT2A-AFF1* (1.1%), and *MEF2D-BCL9* (1.1%). Positive rates of the remaining FGs were all below 1.0% (Fig. 1c).

### FG map of AML

**Fig. 2:**
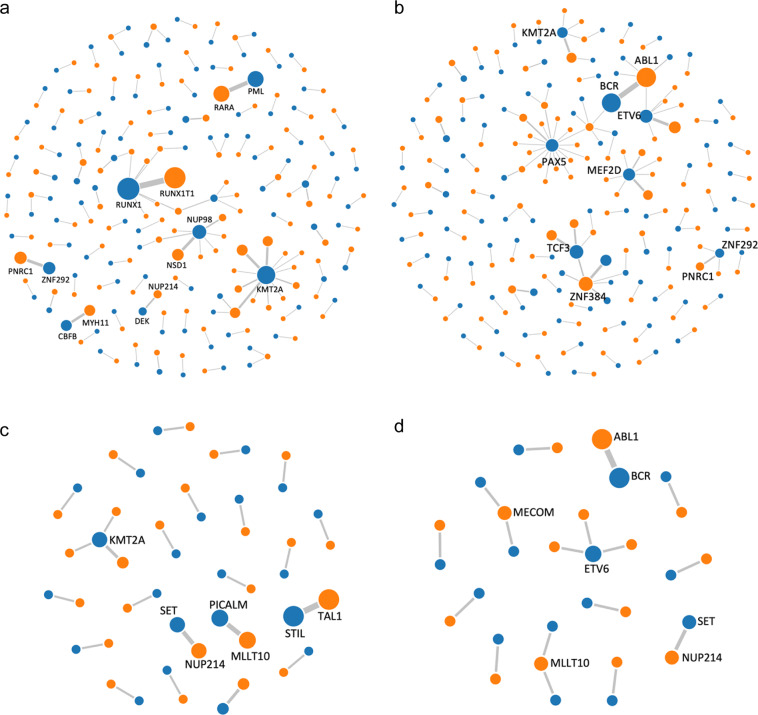
Fusion gene networks in AML, B-ALL, T-ALL, and MPAL. **a** Fusion gene network in AML. **b** Fusion gene network in B-ALL. **c** Fusion gene network in T-ALL. **d** Fusion gene network in MPAL. The blue and orange nodes represent the 5′ fusion gene partner and the 3′ fusion gene partner, respectively. Lines represent fusions. The size of nodes and thickness of lines reflect the frequency of the observed fusions.

We detected 50 kinds of tier A FGs, 58 kinds of tier B FGs, and 25 distinct tier C FGs in 322 (59.7%) of the 539 AML cases (Fig. 2a). The FG incidence in pediatric AML cases was 80.3% (110/137) and significantly higher than 52.7% (212/402) in adult AML cases (*P* < 0.001).

The most frequent FG in AML was *RUNX1-RUNX1T1* (15.2%), followed by *PML-RARA* (7.8%), *ZNF292-PNRC1* (4.1%), *NUP98-NSD1* (3.5%), *CBFB-MYH11* (3.0%), *KMT2A-MLLT4* (3.0%), *KMT2A-MLLT3* (2.6%), *KMT2A-MLLT10* (2.0%), and *DEK-NUP214* (1.3%). Positive rates of the remaining FGs were all below 1.0%. Among them, *RUNX1-RUNX1T1*, *NUP98-NSD1*, *KMT2A-MLLT3*, and *DEK-NUP214* were more frequent in pediatric AML, while *PML-RARA* and *CBFB-MYH11* were more common in adult AML (Table S2).

### FG map of ALL

Within the ALL group, 69 kinds of tier A FGs, 61 kinds of tier B FGs, and 29 distinct tier C FGs were detected in 282 (64.5%) of the 437 ALL cases (Fig. 2b, c). The incidence of FGs in adult ALL was 72.2% (130/180) and significantly higher than 59.1% (152/257) in pediatric ALL cases (*P* = 0.006). The incidence of FGs in B-ALL cases was 67.4% (246/365) and significantly higher than 50.0% (36/72) in T-ALL cases (*P* = 0.007).

The most frequent FG in ALL was *BCR-ABL1* (13.5%), followed by *ETV6-RUNX1* (4.3%), *EP300-ZNF384* (3.7%), *TCF3-PBX1* (3.0%), *KMT2A-AFF1* (2.5%), *MEF2D-BCL9* (2.5%), *STIL-TAL1* (1.8%), *TCF3-HLF* (1.8%), *ZNF292-PNRC1* (1.8%), *EBF1-PDGFRB* (1.1%), *PAX5-NOL4L* (1.1%), *PICALM-MLLT10* (1.1%), and *TCF3-ZNF384* (1.1%). Positive rates of the remaining FGs were all below 1.0%. Pediatric ALL had a higher prevalence of *ETV6-RUNX1*, *MEF2D-BCL9*, and *TCF3-HLF*, while *BCR-ABL1* was more common in adult ALL. *STIL-TAL1* and *PICALM-MLLT10* were detected only in T-ALL, while other FGs, except *ZNF292-PNRC1*, were found only in B-ALL (Table S3).

When we focused on *ZNF384*/*ZNF362*-FM, *PAX5*-FM, and *MEF2D*-FM, which were recently reported as new subtypes in B-ALL [4–7, 17], we found that the incidences of these FG-FMs were second only to *BCR-ABL1*, and all exceeded the well-known *ETV6-RUNX1* and *TCF3-PBX1* in ALL (Table S4).

### FGs detected in MPAL

We detected 22 fusion events (including 15 tier A, 2 tier B, and 5 tier C fusion events, respectively) in 14 (63.6%) of the 24 MPAL cases (Fig. 2d). *BCR-ABL1* was detected in 5 patients. Three *ETV6* fusions (*ETV6-ARNT*, *ETV6-NCOA2*, *ETV6-LOH12CR1*), 2 *MLLT10* fusions (*PICALM-MLLT10* and *NAP1L1-MLLT10*), and 2 *MECOM* fusions (*RUNX1-MECOM* and *TRA2B-MECOM*) were detected in 1 case each. *SET-NUP214* and *KMT2A-MLLT4* were found in 2 cases and 1 case, respectively.

### *KMT2A* fusions

*KMT2A*-FM is a large FG-FM that has been systematically studied and reported in acute leukemia, with more than 100 partner genes, and their specific breakpoint regions have been identified [18, 19]. In this study, fusions of *KMT2A* with 14 different partner genes were detected in 76 cases (7.6%), including a novel *KMT2A-CARS* fusion identified in a pediatric T-ALL case (Fig. S1).

*KMT2A-MLLT4* was the most recurrent, with a frequency of 1.9%, followed by *KMT2A-MLLT3* (1.4%), *KMT2A-MLLT10* (1.2%), and *KMT2A-AFF1* (1.1%). The incidences of *KMT2A-MLLT1* (0.6%), *KMT2A-ELL* (0.4%), *KMT2A-MLLT6* (0.2%), and *KMT2A-USP2* (0.2%) were all below 1%. Besides, one case each with *KMT2A-MAML2*, *KMT2A-MLLT11*, *KMT2A-MYO1F*, *KMT2A-SEPT5*, *KMT2A-SEPT6*, and *KMT2A-CARS* were identified (Table S5).

AML cases (*n* = 539) displayed 16 *KMT2A-MLLT4*, 14 *KMT2A-MLLT3*, 11 *KMT2A-MLLT10*, 4 *KMT2A-MLLT1*, 4 *KMT2A-ELL*, and 2 *KMT2A-MLLT6* fusions. Another 6 *KMT2A* fusions (*KMT2A-MLLT11*, *KMT2A-SEPT5*, *KMT2A-SEPT6*, *KMT2A-MAML2*, *KMT2A-MYO1F*, *KMT2A-USP2*) fusions were detected in 1 case each. ALL cases (*n* = 437) displayed 11 *KMT2A-AFF1*, 2 *KMT2A-MLLT4*, and 2 *KMT2A-MLLT1* fusions. Another 3 *KMT2A* fusions (*KMT2A-MLLT10*, *KMT2A-USP2*, *KMT2A-CARS*) were detected in 1 case each.

On the basis of the above distribution, the incidence of FGs in *KMT2A*-FM was significantly higher in AML (10.6%) than in ALL (4.1%) (*P* < 0.001). Five specific fusions account for 86.0% (*KMT2A-MLLT4* 28.1%, *KMT2A-MLLT3* 24.6%, *KMT2A-MLLT10* 19.3%, *KMT2A-MLLT1* 7.0%, and *KMT2A-ELL* 7.0%) of all *KMT2A* fusions in AML. Three specific FGs account for 83.3% (*KMT2A-AFF1* 61.1%, *KMT2A-MLLT4* 11.1%, and *KMT2A-MLLT1* 11.1%) of all *KMT2A* fusions in ALL.

### Protein kinase gene fusions

**Fig. 3:**
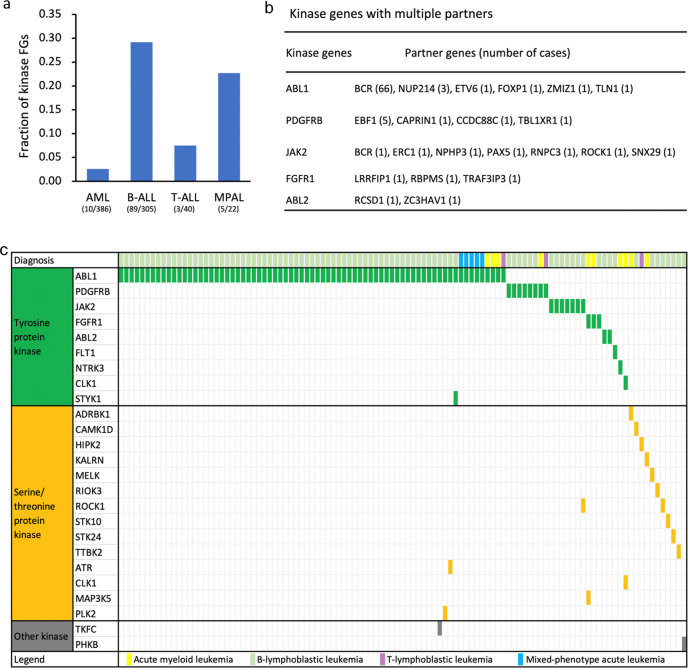
An overview of protein kinase fusions in acute leukemia. **a** Bar plots show the fraction of protein kinase fusions relative to the total number of fusion genes per leukemia subtype. **b** Protein kinase genes with multiple partners. **c** The landscape of protein kinase fusions in different leukemia subtypes. The horizontal and vertical axes represent the patients and kinase genes, respectively. Genes were ordered based on kinase family annotation. Color bar depicts the diagnosis of each sample.

FGs with oncogenic kinase activation have been identified in acute leukemias, and tumor cells harboring these FGs are frequently highly vulnerable to kinase inhibitors [11, 20]. To discover patients who may benefit from targeted kinase inhibitors, we focused on FGs involving a protein kinase gene. Totally, 112 protein kinase fusion events (41 unique fusions) were detected in 107 (10.7%) cases (24.4% in B-ALL, 20.8% in MPAL, 4.2% in T-ALL, and 1.9% in AML). Most protein kinase FGs belonged to the tyrosine kinase family (86.6%). Among them, *ABL*-class (*ABL1*, *PDGFREB*, and *ABL2*) fusions were the most common. *ABL1*, *ABL2*, *PDGFRB*, *JAK2*, *FGFR1*, and *NTRK3* fusions have been reported responsive to targeted kinase inhibitors [20–22], and they were detected in 9.4% of our cases (21.9% in B-ALL, 20.8% in MPAL, 2.8% in T-ALL, and 1.3% in AML) (Fig. 3).

### Transcription factor gene fusions

**Fig. 4:**
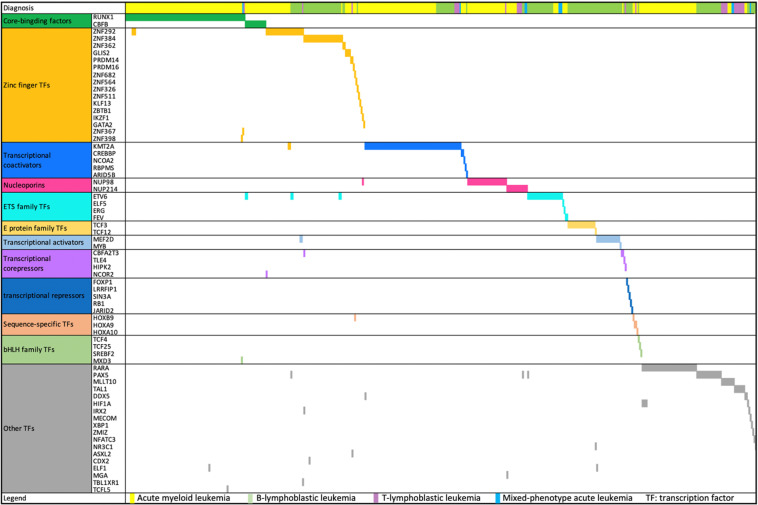
The landscape of transcription factor fusions in different leukemia subtypes. The horizontal and vertical axes represent the patients and transcription factor genes, respectively. Genes were ordered based on transcription factor class. Color bar depicts the diagnosis of each sample.

Chromosomal translocations involving transcription factors are frequently seen in acute leukemia, and some of them have been used as genetic markers for leukemia classification because of their distinctive clinicopathological features and prognostic significance, such as AML with *RUNX1-RUNX1T1* and B-ALL with *TCF3-PBX1*. AML with *RUNX1-RUNX1T1* or *CBFB-MYH11* and acute promyelocytic leukemia with *PML-RARA* are considered to be acute leukemias without regard to blast cell count [12]. In this study, 521 transcription factor-associated FGs (149 distinct fusions) were detected in 485 (48.5%) cases (54.9% in AML, 41.7% in MPAL, 41.6% in B-ALL, and 37.5% in T-ALL). Most of them were core-binding factor (*RUNX1* and *CBFB*) fusions, zinc-finger transcription factor (mainly *ZNF292* and *ZNF384*) fusions, transcriptional coactivator (most of them *KMT2A*) fusions, and nucleoporin (*NUP98* and *NUP214*) fusions (Fig. 4).

### Epigenetic gene fusions

**Fig. 5:**
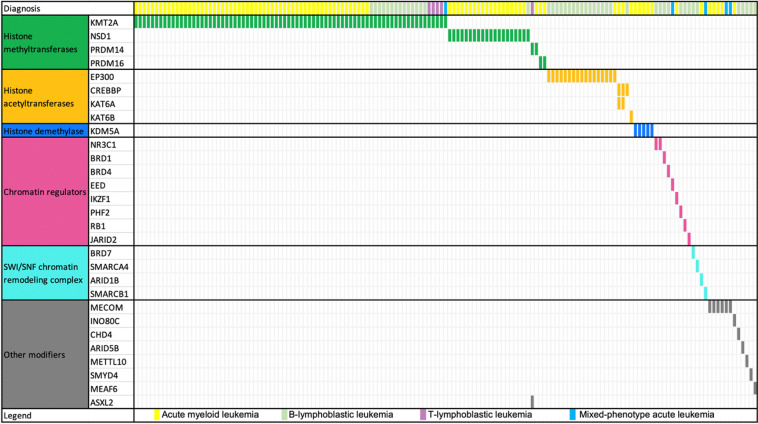
The landscape of chromatin modifier fusions in different leukemia subtypes. The horizontal and vertical axes represent the patients and chromatin modifier genes, respectively. Genes were ordered based on chromatin modifier class. Color bar depicts the diagnosis of each sample.

Recent studies have demonstrated that the genes controlling the chromatin modifier and epigenetic programs include genes that drive human cancer, leading to an increased awareness of the epigenetic protein families as potential drug targets. Inhibitors of DNA methylation and histone deacetylase (HDAC) inhibitors have been approved for clinical use in hematological malignancies, thus providing proof of concept for epigenetic therapies [23, 24]. FGs involving a chromatin modifier and epigenetic gene were detected in 151 (15.1%) cases (20.8% in MPAL, 17.6% in AML, 12.6% in B-ALL, and 6.9% in T-ALL). Most of them (79.6%, 121/152) were histone methyltransferases- or histone acetyltransferases-related FGs. *KMT2A*-related FGs, which may be responsive to *DOT1L* inhibitors [25], were detected in 76 cases (50.3%). *EP300*-related FGs, which may be responsive to the potent, selective *EP300* inhibitor C646 [26], or histone deacetylase inhibitor vorinostat [27], were detected in 17 cases (11.3%) (Fig. 5).

### Novel FGs

Notably, a considerable number of so-far unreported FGs were detected in this cohort. Among the 187 novel fusions (231 fusion events), 13 FGs were detected in 2 or more cases (*ZNF292-PNRC1* in 30 cases; *DDX5-GLUL*, *FOSB-UBC*, and *PTMA-CXCR4* in 3 cases each; the other 9 FGs in 2 cases each), and the other 174 kinds of novel FGs were observed in 1 patient each. The *ZNF292-PNRC1* fusion, which has a prevalence of 3.0% in all cases, was observed in both AML and ALL. The *ZNF292* gene encodes a zinc-finger transcription factor that functions as a tumor suppressor, and eight fusion partners (*B3GAT2*, *CGA*, *FIG4*, *GRM4*, *EYS*, *PRSS54*, *PTPRO*, and *MAP3K4*) have been reported in several solid tumors (invasive breast carcinoma, lower-grade glioma, prostate adenocarcinoma, and sarcoma) in the Tumor Fusion Gene Data Portal. The fusion point of *ZNF292* in all these reported cases was restricted in exon 1, which was the same as in our *ZNF292-PNRC1* positive cases, so we deemed this novel fusion a likely pathogenic FG.

Of the 187 unique novel FGs, 32 (17.1%) were tier A, 98 (52.4%) were tier B, and 57 (30.5%) were tier C FGs. Functional annotation of the genes involved in tier C FGs was performed using the human protein atlas database (https://www.proteinatlas.org), which provides the protein class of the corresponding gene product. A lot of potential functional relevant genes were identified in these FGs, which were annotated as disease-related (*n* = 29), cancer-related (*n* = 18), enzymes (*n* = 17), transcription factors (*n* = 12), transporters (*n* = 12), FDA-approved drug targets (*n* = 8), potential drug targets (*n* = 6), or *RAS* pathway-related proteins (*n* = 2).

**Fig. 6:**
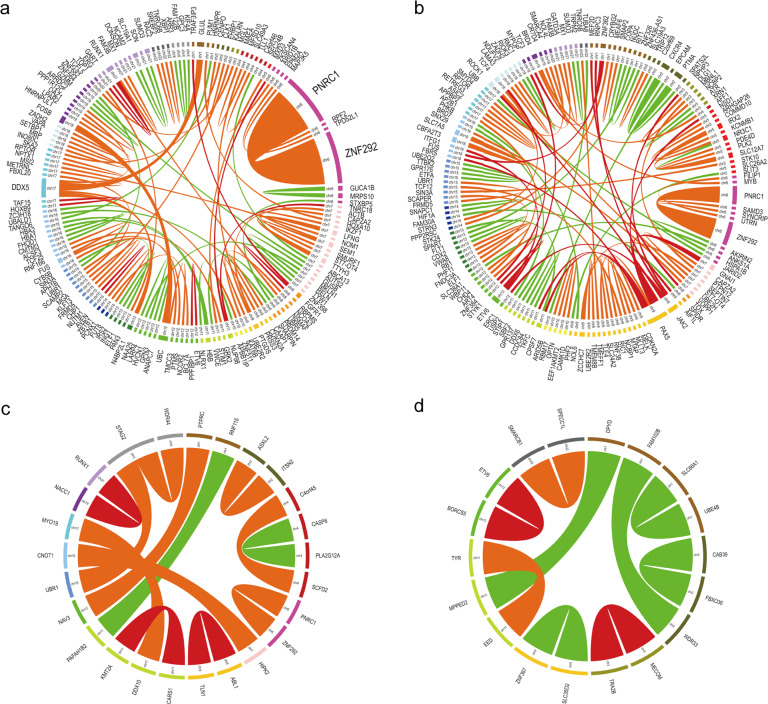
Novel fusion genes detected in AML, B-ALL, T-ALL, and T-ALL. The plot was generated by Circos. The outside circle indicates the chromosomal location of the unique fusion genes. The arcs indicate novel fusions in AML (**a**), B-ALL (**b**), T-ALL (**c**), and MPAL (**d**). The width of the connecting arcs reflects the recurrence of the fusion genes. Different colors of the ribbons indicate tier A (red), tier B (orange), and tier C (green) fusion genes.

In AML, 61.7% (82/133) FGs (9 tier A, 48 tier B, 25 tier C) were to our knowledge not reported before; in B-ALL, 64.2% (88/137) FGs (18 tier A, 45 tier B, 25 tier C) were not reported previously; in T-ALL, 54.2% (13/24) FGs (3 tier A, 7 tier B, 3 tier C) were not described before; and in MPAL, 52.9% (9/17) FGs (2 tier A, 2 tier B, 5 tier C) were not reported before. More than half of novel FGs comprised intra-chromosomal rearrangements (AML: 43/82, 52.4%; B-ALL: 47/87, 54.0%; T-ALL: 7/13, 53.8%; MPAL: 7/9, 77.8%) (Fig. 6).

### Classification of FGs according to FG-FMs

We classified the 230 distinct tier A and tier B FGs according to FG-FMs, which referred to FGs that involve one protagonist gene and various fusion partners. More than half of FGs (119/230, 51.7%) could be classified into 25 FG-FMs, such as *RUNX1*-FM, *KMT2A*-FM, *ABL1*-FM, *RARA*-FM, *ZNF292*-FM, *NUP98*-FM, *ZNF384*-FM, and *ETV6*-FM. The other 111 distinct FGs like *CBFB*-*MYH11*, *CBFA2T3*-*GLIS2*, *EWSR1*-*ELF5*, and *KAT6A*-*CREBBP* could not be classified into any family. Most FGs which could not be clustered into FG-FMs occurred only once. All in all, 77.6% of the 692 tier A and tier B FGs could be classified into FG-FMs. The remaining 22.4% FGs mainly belonged to tier B and rarely recurred in different samples. When we focused on tier A FGs, 94.1% (494/525) could be clustered into FG-FMs, while only 5.9% could not be classified into any FG-FM.

### Comparison of results between WTS and FGs screening

Multiplex-nested RT-PCR, which was designed to detect 41 common FGs (all belonged to tier A FGs), was performed in all 1000 cases, and only 376 (37.6%) cases were positive. All FGs detected by FGs screening were also observed in WTS.

It is worth noting that in 14 cases showing negative results with FGs screening, WTS identified 12 carried *KMT2A* fusions (2 *KMT2A*-*AFF1*, 2 *KMT2A*-*ELL*, 2 *KMT2A*-*MLLT1*, 2 *KMT2A*-*MLLT10*, 2 *KMT2A*-*MLLT6*, 1 *KMT2A*-*MLLT3*, 1 *KMT2A*-*MLLT4*), 1 carried a rare *CBFB*-*MYH11* transcript isoform, and 1 carried a rare *EBF1*-*PDGFRB* transcript isoform. The main fusion isotypes of these FGs have been included in the screening panel, but the variant or rare isoforms conferred negative results.

**Fig. 7:**
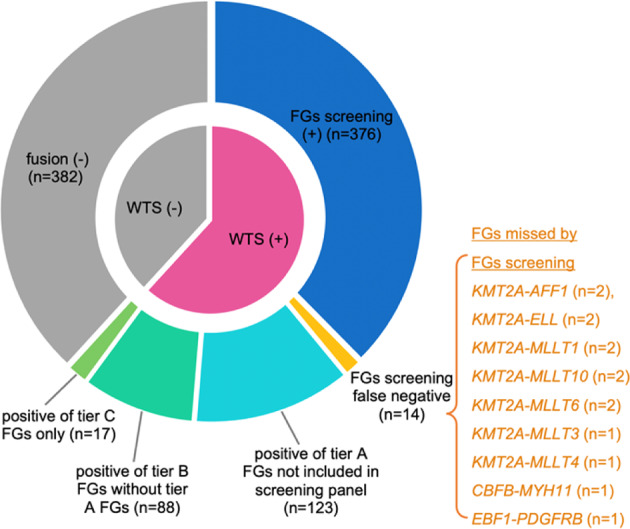
Pie chart represented the comparison of results between whole transcriptome sequencing and fusion genes screening. WTS whole transcriptome sequencing, FG fusion gene.

WTS detected tier A FGs in 513 (51.3%) cases. Therefore, the application of WTS found significantly pathogenic FGs in additional 13.7% cases. Moreover, tier B FGs without tier A FGs were identified in 88 (8.8%) cases, tier C FGs without tier A or B FGs were found in 17 (1.7%) cases (Fig. 7). Although the pathogenicity of tier B FGs needs further clinical and functional verifications, they have a high likelihood that they are pathogenic. Tier C FGs involve many potential functional relevant genes, indicating that some of them may have a potential impact on the pathogenesis of the respective patients.

## Discussion

Recurrent FGs in hematological malignancies are major genetic variants that contribute to tumor genesis. In this cohort, we detected FGs in 61.8% of acute leukemia patients, and the real map of FGs was different from what we expected. Some FGs or FG-FMs may actually have a high incidence but have not been effectively identified before due to cytogenetically cryptic and no observable karyotype abnormalities by conventional chromosome banding analysis. Examples of these previously underestimated FGs include *NUP98*-*NSD1*, both partner genes located close to the telomeric end of chromosome 11 (*NUP98*) and 5 (*NSD1*) [28], or fusions involving *ZNF384*, which is located close to the telomeric end of chromosome 12 [4]. Besides, WTS detected more patients (17.8%) harbored more than one FG, which was detected in 0.3% of patients when detected by limited FGs screening [29]. Mechanism of the concurrence of multiple FGs and the possible cooperative pathogenic mechanism among them merit further study.

The application of WTS could not only reliably detect all FGs revealed by common FGs screening but also found pathogenic (tier A) FGs in an additional 13.7% of cases. Moreover, WTS identified 187 novel FGs in this 1000 cases cohort. More than half of novel FGs comprised intra-chromosomal rearrangements, which are often missed by karyotype analysis. Therefore, WTS proved to be a powerful tool for FGs analysis and has unique advantages for identifying unknown rare or variant FGs. For example, more than 100 *KMT2A* partner genes have been identified, and multiplex-nested RT-PCR methods cannot fully cover them. Moreover, there must be varieties of *KMT2A* fusions that exist but have not yet been identified. Even for the FGs with relatively high incidence, they may also be missed due to the variant breakpoints in *KMT2A*, just as the 12 cases in the present study who had *KMT2A* fusions but showed negative results in FGs screening. Identifying FGs that can be used for sensitive MRD monitoring or with potential therapeutic relevance has critical clinical significance, particularly in cases so far lacking a respective marker or target. For instance, the use of *JAK2* inhibitors might be of use for the patients harboring novel *JAK2* fusions (*ERC1*-*JAK2*, *NPHP3*-*JAK2*, *RNPC3*-*JAK2* [30], *ROCK1*-*JAK2*). In addition, the transcriptional data could provide comprehensive genetic information, including FGs, somatic mutations, tandem duplications, and gene expression. Integrated analysis of these genetic information has the potential to permit precise classification with risk assessment in acute leukemia cases and improve personalized treatment. Based on the experience of implementing WTS as a diagnostic tool in leukemia, Arindrarto et al. [31] showed that WTS is potential to replace all genetic tests for classification and risk assessment of AML except for metaphase cytogenetics.

However, it is worth noting that most nonrecurrent fusion transcripts detected by massively parallel sequencing methods, including WTS, are merely stochastic events and non-pathogenic [32]. How to effectively analyze and differentiate the key FGs from multitudinous background sequences is a great challenge to the analysts’ professional background and analytical capabilities. Attention should also be paid to avoid the false-negative results caused by methodologies or analysis procedures. To better understand the pathological characteristics of FGs, we put forward the conception of FG-FM and classified the final FGs list into four tiers based on our current understanding of their pathogenic impact. FGs in the same FG-FM share commonalities in pathological and clinical features in general, and attention should also be paid to the influence of partner genes on the pathogenicity of FGs. If both FG partners are protagonist genes of one FG-FM, the FG should be classified mainly according to its major pathogenicity and corresponding clinical features. For example, *PAX5*-*JAK2* should be classified under *JAK2*-FM rather than *PAX5*-FM since the gene expression signature and the clinical manifestations of *PAX5*-*JAK2*-positive ALL are similar to *BCR*-*ABL1*-like ALL.

Taken together, WTS has provided a powerful methodology for deciphering the real FG map in acute leukemia comprehensively. Moreover, the map will be increasingly more precise, with the improved ability to analyze the pathological significance of novel FGs and the continuous enrichment of various databases. We described the map of FGs detected in a large cohort of acute leukemias and revealed a considerable number of FGs that have clinical relevance but have not been previously recognized. Classifying FGs according to FG-FMs can help us better understand their pathological significance and suggest new classification patterns for acute leukemia. WTS is a valuable tool and should be recommended in the routine diagnostic workup of acute leukemia.

## Supplementary information

Supplemental figure and tables
