# Supplementary material for: Fusion gene map of acute leukemia revealed by transcriptome sequencing of a consecutive cohort of 1000 cases in a single center
Source: Blood Cancer J. 2021 Jun 16;11(6):112. doi: 10.1038/s41408-021-00504-5 (PMC8209121; doi:10.1038/s41408-021-00504-5)
Supplement: Supplementary file 1 — Supplemental figure and tables [file 41408_2021_504_MOESM1_ESM.pdf]

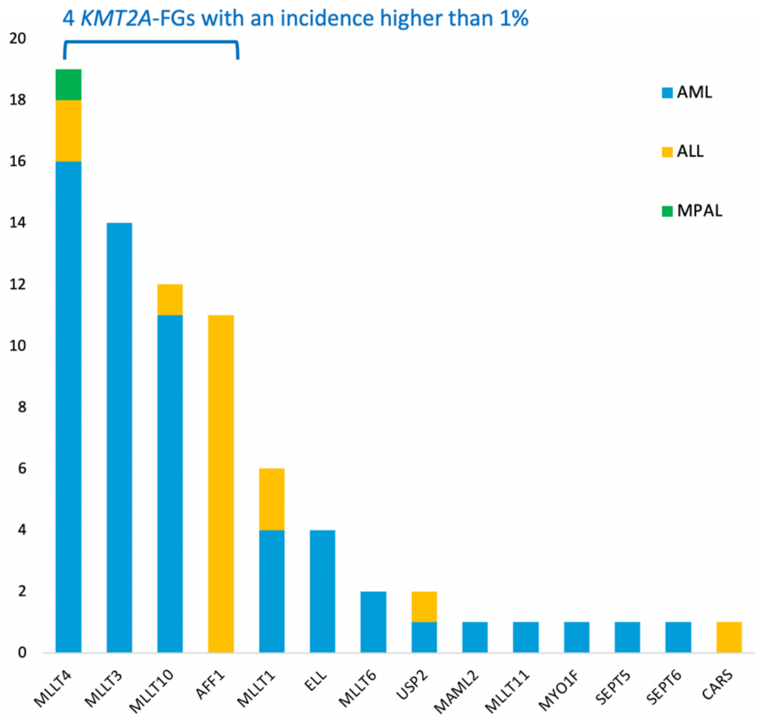

Figure S1. Cases positive of *KMT2A*-related fusion genes. The horizontal and vertical axes represent the different *KMT2A* partner genes identified in 1000 acute leukemia cases and the number of corresponding positive cases. FG, fusion gene.

Table S1. 41 fusion genes and 131 splice variants included in our screening panel

1. *BCR-ABL1* (6)	2. *BCR-FGFR1* (1)	3. *BCR-JAK2* (1)	4. *CBFA2T3-GLIS2* (1)
5. *CBFB-MYH11* (8)	6. *DEK-NUP214* (1)	7. *EBF1-PDGFRB* (1)	8. *ETV6-ABL1* (1)
9. *ETV6-PDGFRB* (1)	10. *ETV6-RUNX1* (2)	11. *FIP1L1-PDGFRA* (5)	12. *FUS-ERG* (5)
13. *KMT2A-AFDN* (4)	14. *KMT2A-AFF1* (12)	15. *KMT2A-ELL* (8)	16. *KMT2A-EPS15* (4)
17. *KMT2A-MLLT1* (4)	18. *KMT2A-MLLT10* (18)	19. *KMT2A-MLLT11* (4)	20. *KMT2A-MLLT3* (8)
21. *KMT2A-MLLT6* (1)	22. *KMT2A-MLLT7* (4)	23. *NPM1-ALK* (1)	24. *NPM1-MLF1* (1)
25. *NPM1-RARA* (2)	26. *NUP98-HOXA9* (1)	27. *NUP98-HOXD13* (1)	28. *NUP98-NSD1* (1)
29. *PAX5-JAK2* (1)	30. *PCM1-JAK2* (1)	31. *PML-RARA* (5)	32. *RUNX1-EAP* (1)
33. *RUNX1-MECOM* (2)	34. *RUNX1-RUNX1T1* (1)	35. *SET-NUP214* (1)	36. *STIL-TAL1* (1)
37. *TCF3-HLF* (3)	38. *TCF3-PBX1* (2)	39. *TCF3-ZNF384* (2)	40. *ZBTB16-RARA* (2)
41. *ZMYM2-FGFR1* (1)			

The numbers in parentheses represent the number of splice variants of each fusion gene.

Table S2. Distribution of positive fusion genes in 539 AML patients

	Total, n (%)	Age, years, n (%)		
		children (≤18)	adult (>18)	*P* value
Total	539 (100)	137 (25.4)	402 (74.6)	
RUNX1-RUNX1T1	82 (15.2)	39 (28.5)	43 (10.7)	<0.001
PML-RARA	42 (7.8)	4 (2.9)	38 (9.5)	0.015
ZNF292-PNRC1	22 (4.1)	2 (1.5)	20 (5.0)	0.082
NUP98-NSD1	19 (3.5%)	11 (8.0)	8 (2.0)	0.002
CBFB-MYH11	16 (3.0%)	0	16 (4.0)	0.016
KMT2A-MLLT4	16 (3.0%)	5 (3.6)	11 (2.7)	0.567
KMT2A-MLLT3	14 (2.6%)	8 (5.8)	6 (1.5)	0.01
KMT2A-MLLT10	11 (2.0%)	4 (2.9)	7 (1.7)	0.483
DEK-NUP214	7 (1.3%)	5 (3.6)	2 (0.5)	0.013

Table S3. Distribution of positive fusion genes in 437 ALL patients

	Total, n (%)	Age, years, n (%)			Immonophenotype, n (%)		
		children (≤18)	adult (>18)	P value	B-ALL	T-ALL	P value
Total	437 (100)	257 (58.8)	180 (41.2)		365 (83.5)	72 (16.5)	
BCR-ABL1	59 (13.5)	11 (4.3)	48 (26.7)	<0.001	59 (16.2)	0	<0.001
ETV6-RUNX1	19 (4.3)	19 (7.4)	0	<0.001	19 (5.2)	0	0.054
EP300-ZNF384	16 (3.7)	6 (2.3)	10 (5.6)	0.118	16 (4.4)	0	0.086
TCF3-PBX1	13 (3.0)	9 (3.5)	4 (2.2)	0.572	13 (3.6)	0	0.139
KMT2A-AFF1	11 (2.5)	6 (2.3)	5 (2.8)	0.766	11 (3.0)	0	0.224
MEF2D-BCL9	11 (2.5)	10 (3.9)	1 (0.6)	0.031	11 (3.0)	0	0.224
STIL-TAL1	8 (1.8)	6 (2.3)	2 (1.1)	0.479	0	8 (11.1)	<0.001
TCF3-HLF	8 (1.8)	8 (3.1)	0	0.023	8 (2.2)	0	0.363
ZNF292-PNRC1	8 (1.8)	6 (2.3)	2 (1.1)	0.479	7 (1.9)	1 (1.4)	0.606

EBF1-PDGFRB	5 (1.1)	1 (0.4)	4 (2.2)	0.164	5 (1.4)	0	1.000
PAX5-NOL4L	5 (1.1)	5 (1.9)	0	0.081	5 (1.4)	0	1.000
PICALM-MLLT10	5 (1.1)	2 (0.8)	3 (1.7)	0.407	0	5 (6.9)	<0.001
TCF3-ZNF384	5 (1.1)	4 (1.6)	1 (0.6)	0.653	5 (1.4)	0	1.000

Table S4. Incidences of *ZNF384/ZNF362*, *PAX5*, and *MEF2D* fusions in B-ALL

FG-FM	Number of patients (%)	FG-FM members (number of cases)
ZNF384/ZNF362-FM	31 (7.1)	*EP300-ZNF384* (16), *TCF3-ZNF384* (5), *TAF15-ZNF384* (4), *EWSR1-ZNF384* (2), *CREBBP-ZNF384* (1), *TCF4-ZNF384* (1), *TCF3-ZNF362* (1), *FUS-ZNF362* (1)
PAX5-FM	21 (4.8)	*PAX5-NOL4L* (5), *PAX5-ZCCHC7* (2), *PAX5-GNAI1* (1), *PAX5-AUTS2* (1), *PAX5-CBFA2T2* (1), *PAX5-CBFA2T3* (1), *PAX5-ELN* (1), *PAX5-ESRRB* (1), *PAX5-FBRSL1* (1), *PAX5-FKBP15* (1), *PAX5-AIF1L* (1), *PAX5-CPSF7* (1), *PAX5-DAK* (1), *PAX5-FBRS* (1), *PAX5-NSD1* (1), *PAX5-EMSY* (1)
MEF2D-FM	20 (4.6)	*MEF2D-BCL9* (11), *MEF2D-HNRNPUL1* (4), *MEF2D-DAZAP1* (1), *MEF2D-FOXJ2* (1), *MEF2D-HNRNPM* (1), *MEF2D-STAT6* (1), *MEF2D-TOX* (1)

Table S5. Distribution of *KMT2A* related fusion genes in different leukemia subgroups

KMT2A-FM	**Total**	AML			B-ALL			T-ALL			MPAL		
		Adult	Children	**Sum**	Adult	Children	**Sum**	Adult	Children	**Sum**	Adult	Children	**Sum**
MLLT4	19	11	5	**16**	0	0	**0**	2	0	**2**	1	0	**1**
MLLT3	14	6	8	**14**	0	0	**0**	0	0	**0**	0	0	**0**
MLLT10	12	7	4	**11**	0	1	**1**	0	0	**0**	0	0	**0**
AFF1	11	0	0	**0**	5	6	**11**	0	0	**0**	0	0	**0**
MLLT1	6	3	1	**4**	0	1	**1**	0	1	**1**	0	0	**0**
ELL	4	3	1	**4**	0	0	**0**	0	0	**0**	0	0	**0**
MLLT6	2	2	0	**2**	0	0	**0**	0	0	**0**	0	0	**0**
USP2	2	0	1	**1**	0	1	**1**	0	0	**0**	0	0	**0**
MAML2	1	0	1	**1**	0	0	**0**	0	0	**0**	0	0	**0**
MLLT11	1	0	1	**1**	0	0	**0**	0	0	**0**	0	0	**0**
MYO1F	1	0	1	**1**	0	0	**0**	0	0	**0**	0	0	**0**
SEPT5	1	1	0	**1**	0	0	**0**	0	0	**0**	0	0	**0**
SEPT6	1	0	1	**1**	0	0	**0**	0	0	**0**	0	0	**0**
CARS	1	0	0	**0**	0	0	**0**	0	1	**1**	0	0	**0**
**Total**	76	33	24	**57**	**5**	**9**	**14**	**2**	**2**	**4**	**1**	**0**	**1**
